# Dynamic regulation of tumour progression by phenotype‐switching drivers

**DOI:** 10.1002/ctm2.840

**Published:** 2022-07-28

**Authors:** Laurenz Vock, Anna Gschwendtner, Oliver Eckel, Madalina A. Mirea, Markus Hengstschläger, Mario Mikula

**Affiliations:** ^1^ Center for Pathobiochemistry and Genetics Medical University of Vienna Vienna Austria

**Keywords:** EMT, epigenetics, proliferation, transcription factors

## 1 BACKGROUND

For decades, researchers have strived to analyze the process of tumour formation and progression. Despite the immense accumulation of knowledge, cancer still remains one of the biggest burdens of our society today. According to the WHO, nearly one in six deaths worldwide was attributed to cancer in 2020. Hence, it is crucial to revisit and build upon recent achievements to improve our understanding of this disease.

One of the most important concepts to describe the growth and spreading of tumour cells in patients over time is the phenotype‐switching model.^1^ This model was first discovered in melanoma and is also applicable to other tumours. It is based on the idea that tumour cells can exist in two different cellular states. On the one hand, they exhibit a high proliferative activity with a high rate of glycolysis, but low migrative and invasive capacity. On the other hand, cells can exist in a state hallmarked by up‐regulation of migrative and invasive behaviour with inflammation‐related pathways being increased. Therefore, increasing evidence supports the idea that tumours progress to metastasis by dynamically switching from a local, proliferative state to a highly migrative state, which is later converted back to a proliferative state once the site for metastasis formation has been reached. This model elegantly described the observed spread of cancer in patients, but key drives, which are instrumental for inducing the switch from one phenotype to another, are still ill defined. Epigenetic regulators in concert with activation of transcription factors are likely involved in this process. Therefore, important novel aspects on the regulation of phenotype switching are summarized below.

## 2 COMMENTARY

Chromatin remodelling enzymes comprise DNA methyltransferases, histone deacetylases and histone methylases. The latter have emerged as important players during cancer progression, and with much interest we have read a recently published article on the role of KDM4C in prostate cancer.^2^ KDM4C is a nuclear protein that converts trimethylated histone residues to the dimethylated form and has been implicated in regulating cancer proliferation.^3^ Interestingly, Lin et al. identified regulation of the MYC transcription factor after modulating KDM4C amounts and specifically showed that overexpression of KDM4C‐induced MYC promoter activity. On the contrary, knockdown of KDM4C, in two prostate cell lines, resulted in decreased MYC expression. Knockout of KDM4C reduced the metabolic potential of cells, as well as gene expression of GAPDH, HK2 and LDH, indicating a decrease in glycolysis. Functionally, KDM4C knockout decreased cancer progression in a zebfrafish xenotransplantation model.

The MYC transcription factor is a significant driver of cancer growth and is known to regulate glucose uptake, lipid synthesis and polyamine synthesis.^4^ In the below‐mentioned model, MYC represents a proponent for increased cellular proliferation and increased aerobic glycolysis. Hence, the identification of MYC as a target of KDM4C activity is an important step in revealing drivers of phenotype switching.

Another interesting histone methylaseis KDM5B, also termed JARID1B, is crucial for inducing an epithelial–mesenchymal–transition of cancer cells.^5^ This process is hallmarked by up‐regulation of E‐cadherin suppressors, such as SNAI1, TWIST1 and ZEB1, as well as acquisition of a migrative and invasive phenotype. As cells shift from expression of pro‐proliferative transcription factors to EMT promoting transcription factors, cellular behaviour including cell cycle and metabolism also changes.

Furthermore the epigenetic modulator PHF8, also called KDM7B, has been found to directly control TGF beta signalling, which is essential for the EMT process.^6^ Importantly, loss of PHF8 was associated with decreased cell invasion without affecting proliferation.

**FIGURE 1:**
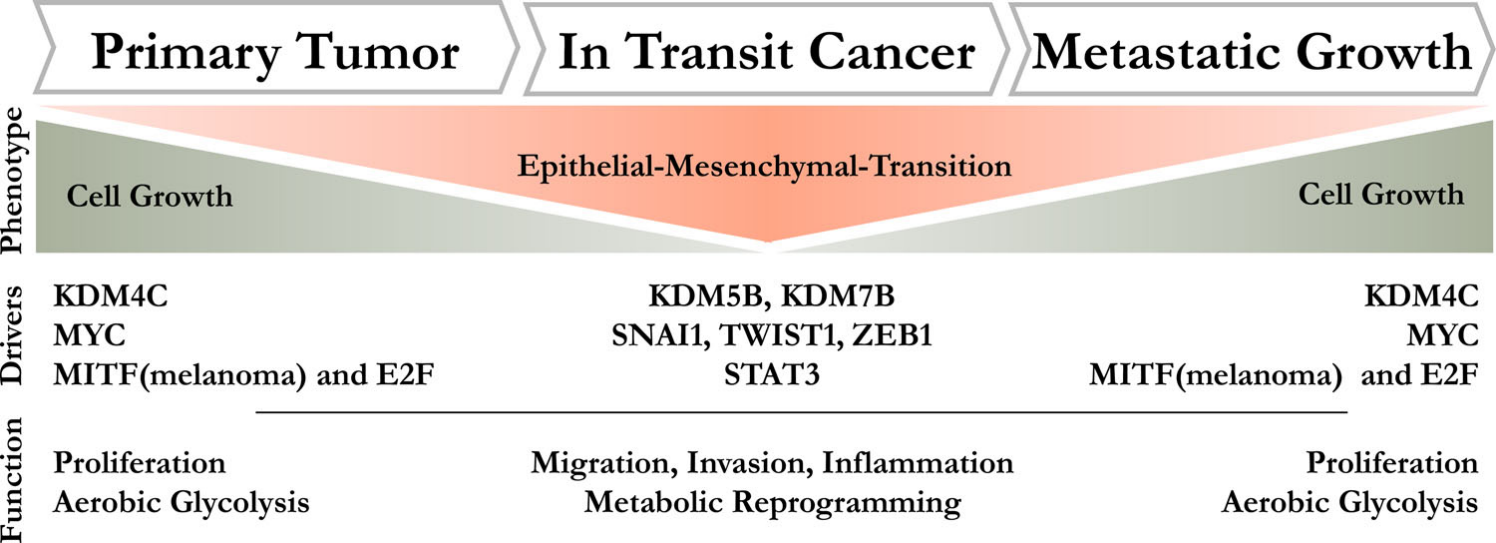
Phenotypic changes during cancer progression. Primary tumours are hallmarked by strong cellular growth. Malignancy progresses when migrative and invasive behaviour is gained and tumour cells colonize distant organs, where tumour growth is resumed. Concomitantly, histone modification enzymes like KDM4C, KDM5B and KDM7B(PHF8) are involved in this process and may contribute to regulation of distinct transcription factors, which in turn are associated with defined functional states

This points to another interesting Yin and Yang regulation phenomenon on the level of transcription factors. STAT3 often works in concert with TGF beta and has been shown to be crucially involved in cancer cell dissemination. Evidence suggests that STAT3 can suppress expression of MITF, which is one of the main factors driving melanoma proliferation and differentiation.^7^ Hence, this antagonistic regulation, again, demonstrates the presence of distinct cellular states (Figure 1).

## 3 CONCLUSION

From these examples, we can learn that developmental forces exist, which lead to differentiation or de‐differentiation of cancer cells. These changes are either induced or accompanied by expression of epigenetic modulators, which result in a change in expression of transcription factors. Since those transcription factors are usually not amendable for therapeutic targeting, it is much more compelling to target the epigenetic regulators in order to achieve beneficial outcome. Members of the KDM family of proteins could be considered as the switching drivers in various cancers and should therefore be closely investigated in order to develop and test novel inhibitory compounds.

Finally, tumour therapy is most efficient when a combinatory drug treatment approach regimen is pursued. Blocking invasive capabilities together with the proliferative potential, as outlined here, seems to be a very promising approach.

## CONFLICT OF INTEREST

The authors declare no conflict of interest.
